# Clinical and epidemiological analysis of *Campylobacter fetus* subsp. *fetus* infections in humans and comparative genetic analysis with strains isolated from cattle

**DOI:** 10.1186/s12879-016-1538-7

**Published:** 2016-05-14

**Authors:** Robert Escher, Colette Brunner, Niklaus von Steiger, Isabelle Brodard, Sara Droz, Carlos Abril, Peter Kuhnert

**Affiliations:** Institute of Veterinary Bacteriology, Vetsuisse Faculty, University of Bern, Bern, Switzerland; Department of Medicine, Spital Emmental, Burgdorf, Switzerland; Institute for Infectious Diseases, University of Bern, Bern, Switzerland; Current address: Institute of Virology and Immunology, University of Bern, Bern, Switzerland

**Keywords:** *Campylobacter fetus* subspecies *fetus*, Epidemiology, Multilocus sequence typing (MLST), Pulsed-field gel electrophoresis (PFGE), Major outer membrane protein (MOMP), Type IV secretion system (T4SS)

## Abstract

**Background:**

*Campylobacter fetus* subspecies *fetus* (CFF) is an important pathogen for both cattle and humans. We performed a systematic epidemiological and clinical study of patients and evaluated the genetic relatedness of 17 human and 17 bovine CFF isolates by using different genotyping methods. In addition, the serotype, the dissemination of the genomic island containing a type IV secretion system (T4SS) and resistance determinants for tetracycline and streptomycin were also evaluated.

**Methods:**

The isolates from patients diagnosed with CFF infection as well as those from faecal samples of healthy calves were genotyped using pulsed-field gel electrophoresis (PFGE), multilocus sequence typing (MLST), as well as single locus sequence typing (SLST) targeting *cmp1* and *cmp2* genes encoding two major outer membrane proteins in CFF. The presence of the genomic island and identification of serotype was determined by PCRs targeting genes of the T4SS and the *sap* locus, respectively. Tetracycline and streptomycin resistance phenotypes were determined by minimal inhibitory concentration. Clinical data obtained from medical records and laboratory data were supplemented by data obtained via telephone interviews with the patients and treating physicians.

**Results:**

PFGE analysis defined two major clusters; cluster A containing 16 bovine (80 %) isolates and cluster B containing 13 human (92 %) isolates, suggesting a host preference. Further genotypic analysis using MLST, SLST as well as *sap* and T4SS PCR showed the presence of genotypically identical isolates in cattle and humans. The low diversity observed within the *cmp* alleles of CFF corroborates the clonal nature of this pathogen. The genomic island containing the tetracycline and streptomycin resistance determinants was found in 55 % of the isolates in cluster A and correlated with phenotypic antibiotic resistance.

**Conclusions:**

Most human and bovine isolates were separated on two phylogenetic clusters. However, several human and bovine isolates were identical by diverse genotyping methods, indicating a possible link between strains from these two hosts.

## Background

The species *Campylobacter fetus* is divided into two subspecies, *Campylobacter fetus* subspecies *fetus* (CFF) and *Campylobacter fetus* subspecies *venerealis* (CFV). CFV is host restricted and is the causative agent of bovine genital campylobacteriosis. In contrast, CFF is a pathogen affecting animals as well as humans. It has commensal characteristics in a wide range of animals and is most often associated with abortion in sheep and to a lesser extent in cattle [1]. In humans, CFF is recognized within other *Campylobacter* species as a major causative factor in extraintestinal illness. It has emerged as a human pathogen responsible for serious systemic disease, and although responding mostly to ampicillin, gentamicin and carbapenems [2], there is still a documented mortality rate of up to 14 % [3]. CFF has a predilection for the human vascular endothelium, and the majority of reported infections include blood stream infections and haematogenous spread. CFF has been isolated from blood or stool of humans with endocarditis, mycotic aneurysms, cellulitis, abscesses, meningitis or meningoencephalitis, vertebral osteomyelitis, septic arthritis, spontaneous bacterial as well as continuous ambulatory peritoneal dialysis associated peritonitis, infectious diarrhea, and more rarely, with pelvic infection, cholecystitis, pericarditis, pleurisy, pneumonia, urinary tract infection and abortion [3–13]. CFF mostly affects immunocompromised patients, elderly people with an underlying disease and neonates. The apparently emerging status of CFF might be explained by the improvement of diagnostic methods, increased medical awareness and the growing number of immunocompromised patients [3, 6–13].

Identification of animal species serving as reservoirs for human infection and detection of routes of transmission remain a major public health issue. It is hypothesized that exposure to livestock and raw or undercooked animal products represent the main sources for human CFF infections [6]. CFF has been identified in faeces of beef cattle, sheep, pigs, monkeys, and horses [6, 14, 15]. However, so far no comparative analysis of geno- and phenotypic data of human and animal CFF strains has been performed in a given geographic region, and data from epidemiologic studies of human CFF infection are still scarce. In the case of CFF, the differentiation of *Campylobacter fetus* subspecies has been challenging [16] with reliable techniques only established recently.

A MLST schema for *C. fetus* has previously been reported [14]. A strong correlation was observed between MLST and PFGE data as well as between MLST and the two serotypes defined by *sapA* and *sapB*. This study demonstrated the clonal nature of the *C. fetus* species and suggested the MLST typing method as the most appropriate procedure for long-term epidemiological and phylogenetic studies.

In order to increase the discriminatory power and reliably trace the source and route of transmission, further strain characterization is helpful. Bacterial outer membrane proteins of Gram-negative bacteria are considered the major mediators of the pathogen-host interaction and are essential for the microbial adaptation to the host environment. The *porA* gene encoding the major outer membrane protein (MOMP) of *C. jejuni* and *C. coli* has been used to enhance genetic discrimination of isolates and to identify epidemiologically linked campylobacter isolates [17]. In *C. fetus*, the presence of 2 MOMP with porin activity but different from PorA has been demonstrated, one encoded by *cmp1* (GenBank locus tag CFF8240_0396) and the other by *cmp2* (GenBank locus tag CFF8240_0397), prompting us to include these in our analysis. Furthermore, T4SS are multicomponent transporters of Gram-negative bacteria with functions as diverse as the delivery of effector proteins into eukaryotic cells in pathogenesis or DNA transfer in bacterial conjugation [18]. T4SS associated to genomic islands are crucial for the horizontal transfer of these genomic islands and might disseminate antibiotic resistance elements [19]. We have recently described the presence of a genomic island in CFF carrying a T4SS and two genes conferring resistance to tetracycline and streptomycin [20].

In this study, we conducted a comprehensive analysis of a large number of CFF isolates from humans and animals in order to thoroughly investigate the epidemiology of infection in humans as well as the genetic diversity of bacterial strains and the potential role of cattle as a reservoir for human CFF infections. Seventeen strains isolated from humans with CFF infection and 17 strains from bovine carriers were analyzed by PFGE, MLST, *sap*/serotyping and by further genotyping based on *cmp1/cmp2* sequences and PCR detection of T4SS. In addition, antibiotic resistance towards tetracycline and streptomycin was determined phenotypically using minimal inhibitory concentration (MIC).

## Methods

### Bacterial isolates

Bacterial CFF isolates from 17 patients were identified between 2001 and 2009 at our institution, a university hospital in Switzerland with specialized microbiological laboratories serving an estimated population of 1.2 million. Confirmation of human CFF infections was based on positive culture results. *C. fetus* was identified by sequencing 500 bp of the 16S rRNA gene. Clinically relevant patient information was obtained from medical charts and supplemented by telephone interviews with patients and treating physicians. Seventeen bovine isolates originated from faecal samples of healthy calves from different geographic regions of Switzerland. Bacterial strains were cultured on tryptone soya agar containing sheep blood 5 % v⁄v (Oxoid, Wesel, Germany) under microaerobic conditions for 2 days at 37 °C. *C. fetus* subsp. identification was based on glycine tolerance test. The results were confirmed by PCR [21].

### Geno- and phenotyping, cluster analysis

PFGE was performed using the restriction enzyme *Sma*I as previously described [21, 22]. The DNA banding pattern was analyzed using BioNumerics software (version 7.5; Applied Math, Sint-Martens-Latem, Belgium) and a dendrogram was generated by cluster analysis using a similarity matrix (settings: Dice, UPGMA with 3 % optimisation and 1 % band tolerance). A cut-off at 70 % similarity of the Dice coefficient was applied to indicate major PFGE clusters. Fisher’s exact test was used for statistical analysis of the dendrogram.

The 2 MOMP encoding genes *cmp1* and *cmp2* in CFF were partially sequenced. Primers were designed based on the reference genome sequence of *C fetus* subsp*. fetus* 80–40 (GenBank acc. no. NC_008599) to amplify fragments encoding the C-terminal part of these proteins which contain putative external loop structures, and are predicted to be the most variable regions in these proteins. The 3′ part of the gene *cmp1* was amplified with primer pair CFMOMP1_2-L (5′-TTGAAAATGTTACTGATCTATACGCTATAGAC) and CFMOMP1-R (5′-AGTTTTATCTTTACCAGCATTGTCTGGCTCTT CAGTT) resulting in a 536 bp PCR product (position 650–1185 of *cmp1*). The 3′ part of *cmp2* was amplified with primer pair CFMOMP1_2-L and CFMOMP2-R (5′-GTTTTTGAAATCGTTTTTGCCATCTATC) resulting in a 545 bp PCR product (position 641–1185 of *cmp2*). Sequence alignments were performed using the Vector NTI advance software (Invitrogen), and a unique arbitrary number was given to every single allele based on the reference sequences of *cmp1* and *cmp2* of strain 80–40 defining allele number 1 (GenBank locus tag CFF8240_0396 and CFF8240_0397, respectively). The four allele sequences of *cmp1* and the five of *cmp2* have been deposited at GenBank under accession numbers KU670818-KU670821 and KU670822-KU670826, respectively. A full matrix including sequences of all strains analyzed in this study can be accessed at http://purl.org/phylo/treebase/phylows/study/TB2:S18853.

The MLST analysis was carried out using the protocols of the *C. fetus* MLST database as described before [14]. New alleles and MLST sequence types (ST) were deposited in the PubMLST database (http://pubmlst.org/cfetus/). PCR screening for the presence of the T4SS and susceptibility tests determining MIC for tetracycline and streptomycin were carried out as previously described [20]. The analysis of the *sap*/serotypes was done by PCR as published [23].

## Results

### Clinical and epidemiological data of human patients

In humans, diagnosis of CFF infection was based on positive blood cultures in 76.5 % of cases, and on other sources in 41.1 % of cases (stool, abscess fluid, biopsies; in addition to blood cultures in cases 1, 9, 12; Table 1). CFF was mostly isolated from elderly patients (mean age, 65 years; range 31–87 years), with a slight preponderance of male patients (10 males, 7 females). The site of infection was the gastrointestinal tract (colitis) in 6 cases, the native venous tract in 4 cases (deep venous thrombosis in a leg, in 1 patient (case 11) associated with erysipelas), the skin (erysipelas) in 2 cases, the site of foreign material (3 joint prostheses, 1 vascular Y-prosthesis, 1 implanted central venous catheter Port-a-Cath®) in 5 cases, and in 1 patient the urinary tract. Abscess formation was noted in 3 cases: 2 psoas abscesses associated either with colitis (case 9) or with infection of the hip prosthesis (case 15), and 1 abscess in the deep tissues of the plantar foot associated with thrombosis in the leg (case 12). Of the 6 patients with a joint prosthesis, only 3 had the infection at the site of the implant. Seven patients were immunocompromised, confirming previous observations for susceptibility to CFF infection. Underlying diseases included most commonly heart disease (9 patients), arterial hypertension (8 patients), renal failure (5 patients) and type 2 diabetes mellitus (4 patients), underscoring the morbidity of this mostly elderly population. Interestingly, the presence of diverticulosis (of the sigmoid colon in patients 2, 13 and 15, and of the whole colon in patients 3, 14 and 17) did not predispose to gastrointestinal infection with CFF, as only patients 14 and 17 became symptomatic, whereas colitis was a presenting symptom in patients 1, 4, 6 and 9 who did not have known diverticulosis. All but patients 10, 11 and 12 were moderately anaemic (82 %, haemoglobin 90–110 g/L). We found two co-infections: in patient 1 with *Clostridium difficile* and in patient 9 with *Campylobacter jejuni* (both isolated from stool specimens). Possible bacterial entry routes were skin or mucosal lesions in at least 14 out of the 17 patients; for patient 2, this information was not available. Skin lesions were open wounds including a leg ulcer (patient 11), whilst mucosal lesions included open wounds after extraction of a wisdom tooth and removal of a mandibular cyst (patient 1), oral ulcers (patient 5), ulcerous cavities due to poor dental status (patient 9), erosive gastritis (patient 7) and oesophagitis (patient 4).

**Table 1:** Patient data

case	Bacterial strain number	Site of infection, symptom	Sample(s) for analysis	Comorbidities	Immuno-suppression	Skin/mucosa lesion	Joint prosthesis	Ingestion of raw food	Animal contact	Therapy	Outcome
1	H_900686	colitis	blood, stool	KT, HT, RF	yes (pt)	yes (enoral)	no	v	dogs	Q	recovery
2	H_914559	UTI	blood	KT, HD, Div, RF	yes (pt)	u	hip	v, e	cats	Q	recovery
3	H_932845	erysipelas	blood	HD, HT, DM, Div, W	yes (st, cy)	no	no	u	u	P	recovery
4	H_1029625	colitis, VT	blood	AIDS, HD, COPD	yes (AIDS)	yes (esophageal)	no	rm, e	no	Cla	recovery
5	H_1103393	elbow joint prosthesis	biopsy gt elbow joint	RA	yes (chloroquine)	yes (elbow, enoral)	elbow	rm	dogs, horses	P; Q, R	relapse 2 years later
6	H_1277769	colitis	blood	LT, PV, RF, HT	yes (pt)	no	no	v	no	Q	recovery
7	H_1113346	Port-a-Cath	blood	NHL	yes (chemotherapy)	yes (skin, erosive gastritis)	no	v, e	dogs	Q	recovery
8	H_1084325	VT	blood	HD, anemia	no	yes (skin)	no	no	no	P	recovery
9	H_1136201	colitis, psoas abscess	abscess, blood	HD, COPD, HT, Dem, AlcAb	no	yes (mucosa)	hip	rm, e, g	no	Q	recovery
10	H_1223747	hip joint prosthesis	biopsy gt hip joint; stool	-	no	yes (esophageal)	hip	v, rm	cats, dogs	Q, R; Cla; Q	chronic infection
11	H_1289205	VT, erysipelas	blood	HD, DM	no	yes (skin)	no	v	c, dogs, poultry	P; C	recovery
12	H_1437517	VT, abscess foot	biopsy gt and abscess, foot, blood	HT, portacath	no	yes (skin)	hip, knees	v	c, cats, p, dogs	P; C; Q	recovery
13	H_1456372	vascular Y-prosthesis	blood	HT, HD, RF, DM, Div	no	yes (mucosa)	no	v, e, g	p	C; Do	recovery
14	H_1473712	colitis	stool	HT, HD, Div, DM, COPD	no	yes (mucosa)	no	rm	no	Cla	recovery
15	H_1516477	hip joint prosthesis, psoas abscess	abscess fluid; biopsy gt hip	Div	no	yes (mucosa)	hip	v	s, cats	Q; P	recovery
16	H_1539713	erysipelas	blood	HT, HD, RF, PD	no	yes (skin)	no	v	no	P	recovery
17	H_1599086	colitis	blood	Div, COPD, AlcAb	no	yes (mucosa)	no	v	bi, tu, fi	Q	recovery

*AIDS* acquired immunodeficiency syndrome, *AlcAb* alcohol abuse, *COPD* chronic obstructive pulmonary disease, *Dem* dementia, *Div* diverticulosis, *DM* diabetes mellitus type 2, *HD* heart disease, *HT* hypertension, *KT* kidney transplant, *LT* liver transplant, *PD* Parkinson’s Disease, *PV* polycythemia vera, *RA* rheumatoid arthritis, *RF* renal failure, *UTI* urogenital tract infection, *VT* venous thrombosis, *W* Wegener granulomatosis, *gt* granulomatous tissue, *cy* cyclophosphamid, *pt* post-transplant, *st* steroids, *e* raw eggs, *rm* raw meat, *u* unknown, *v* vegetables, *bi* birds, *c* cattle, *fi* fish, *g* goat cheese, *gp* guinea pigs, *p* pigs, *s* sheep, *tu* turtles, *C* carbapenem, *Cla* clarithromycin, *Do* doxycycline, *P* amoxicillin/clavulanic acid, *Q* quinolone, *R* rifampicin

Most of the patients (59 %) were treated with quinolones (ciprofloxacin or ofloxacin), especially when gastrointestinal symptoms were present. In the case of colitis, clarithromycin was successfully used (patients 4 and 14). In the cases of soft tissue infections (erysipelas, abscess), either in the presence or absence of venous thrombosis, amoxicillin was more readily used. The use of antibiotics was always adapted to the susceptibility results of the isolates. In the patients with prosthetic device infection (joints and catheter), only complete removal of the synthetic material and concomitant, longstanding antibiotic therapy led to the resolution of the infection. For example in patient 15, symptomatic CFF infection recurred 6 years after the first CFF infection and antibiotic therapy; the patient was finally cured after removal of the osteosynthetic material. In patients 5 and 10, antibiotic therapy alone over years (2 and 3 years, respectively) led to control of the chronic infection and formation of a fistula.

At the time of CFF bacteremia, patient 13 had a PET/CT scan showing ^18^F-FDG uptake at the site of the aortoiliac Y-prosthesis, suggesting infection of the prosthesis which was implanted 2 years before. Antibiotic therapy alone (meropenem followed by oral doxycycline for 3 months) led to resolution of the inflammatory signs in the blood and negative blood cultures on follow-up. None of the 17 patients died due to CFF infection.

Epidemiological data reveal contact with animals for 59 % of the patients. However only a minority had contact with animals known to be carriers of CFF: horses (patient 5), cattle (patient 11 and 12), pigs (patients 12 and 13), sheep (patient 15) and turtles (patient 17). All but one subject (patient 8) either consumed raw or undercooked meat (patients 4, 5, 9, 10, 14), raw eggs (patients 2, 4, 7, 9, 13) and/or raw vegetables (patients 1, 2, 6, 7, 10, 11, 12, 13, 15, 16, 17). Patient 13 was a butcher, and patients 1, 2, 3, 5, 8, 9, 10, 11, 12, 13, 15, and 16 (71 %) were regularly visiting farms or were working there (patients 11, 12 and 15 were farmers). One patient (case 6) frequented a swimming pool, and only 2 patients (cases 10 and 15) had contact with young children. Traveling was limited: 9 patients never traveled, 5 patients visited other small localities within Switzerland occasionally, and 3 patients had been to Southern Germany, Spain, or South Africa in the last 3 years.

### Comparative genotypic and phenotypic analyses between human and bovine isolates

#### PFGE, MLST and sap/serotyping

**Fig. 1:**
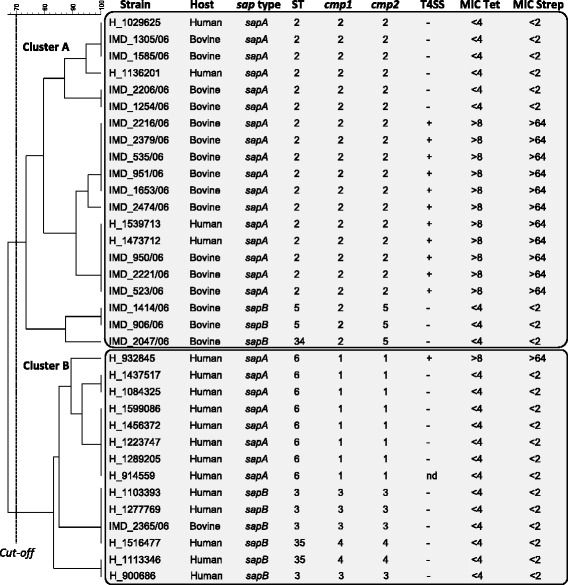
Cluster analysis of all human and bovine strains. A dendrogram was generated using a similarity matrix (settings: Dice, UPGMA with 3 % optimisation and 1 % band tolerance) from the results of pulse-field gel electrophoresis using the restriction enzyme *Sma*I and DNA banding analysis using BioNumerics software. Cluster analysis was completed with the genotypic and phenotypic characteristics of the strains: multilocus sequence typing (ST), *sap*/serotype, allele derived from gene sequences for major outer membrane proteins *cmp1* and *cmp2*, presence (+) or absence (−) of a genomic island containing type IV secretions system (T4SS) and resistance (expressed as minimal inhibitory concentration, MIC) to the antibiotics tetracycline (MIC Tet) and streptomycin (MIC Strep). With a cut-off at 70 % of similarity of the Dice coefficient, 2 major clusters A and B are identifiable

PFGE analysis grouped the human and bovine CFF isolates into two major clusters A and B (Fig. 1). These two main clusters showed a Dice similarity coefficient higher than 70 %. In agreement with previous observations [14], there was a 100 % correlation between results of PFGE and MLST analyses. Cluster A contained 17 isolates of the MLST sequence type (ST) ST-2, 2 isolates of ST-5, and 1 isolate of ST-34. Cluster B contained 8 isolates ST-6, 4 isolates ST-3, and 2 isolates ST-35. There was no overlap of ST between the clusters.

Serotyping by PCR gave a specific band with all isolates, either for *sapA* or for *sapB* but never for both. The two serotypes were found in both clusters and in human as well as bovine isolates. There was an association of serotypes with subclusters in cluster A and B determined by PFGE as well as MLST (Fig. 1).

Cluster A contained 4 human and 16 bovine isolates, and cluster B 13 human and 1 bovine isolate. Within cluster A, of the 4 isolates originating from human samples, 1 and 2 isolates were sharing a 100 % homology of the analyzed characteristics with 2 and 3 isolates from cattle, respectively (human isolate H_1029625 with cattle isolates IMD_1305/06 and IMD_1585/06; human isolates H_1539713 and H_1473712 with cattle isolates IMD_950/06, IMD_2221/06 and IMD_523/06). Within cluster B, the only strain isolated from cattle shared characteristics with 2 strains isolated from humans (cattle isolate IMD_2365/06 and human isolates H_1103393 and H_1277769). Overall, a striking separation of strains from bovine and human origins into clusters A and B, respectively, was observed, indicating an association between strain and origin of isolates (*p* < 0.0001). Despite this fact, identical strains between human and bovine samples were identified in each cluster.

### cmp1 and cmp2 analyses

The locus tags CFF8240_0396, and CFF8240_0397 of the CFF strain 82–40 (GenBank acc. No: NC_008599) were used as a reference for *cmp1* and *cmp2*, respectively. The nucleotide diversity of the 3′ part of both *cmp* genes was analyzed in all the isolates, and arbitrary numbers setting the reference sequence as 1 were used to distinguish the allele sequences (Table 2). Fifteen and 22 variable nucleotide positions were identified in *cmp1* and *cmp2*, respectively. Silent substitutions are indicated in lower cases, non-silent ones in upper cases. They were calculated using the in frame predicted amino acid sequences of both genes. Interestingly, some of the nucleotide substitutions are shared between both, equally long *cmp* gene sequences, indicating most likely a gene duplication event and/or the same purifying selection. The allele type of both genes correlated highly with the clusters obtained by PFGE and MLST, and a similar level of discrimination was observed as for the PFGE and MLST analyses (Fig. 1). Moreover, *cmp* gene sequence types demonstrate a 100 % discrimination between cluster A and B. With *cmp1* only type 2 was found in cluster A, i.e. in the human as well as the bovine isolates. In cluster B, only *cmp1* sequence types 1, 3, and 4 were found. Type 3 was found in the bovine as well as in human isolates. With respect to *cmp2*, sequence types 2 and 5 were only found in cluster A, with 2 found in both, human and bovine, while 5 only in bovine isolates. Types 1, 3 and 4 were only detected in cluster B with type 3 in isolates from both hosts.

**Table 2:** Variable nucleotide positions identified within the 3′ part of *cmp1* and *cmp2* of CFF

Gene/Allele^a^	Nucleotide substitutions and position within the gene																					
*cmp1*	688	696	697	712	715	716	720	723	726	729	732	738	740	741	825	960						
**1**	**G**	**T**	**G**	**T**	**A**	**A**	**T**	**A**	**G**	**C**	**A**	**C**	**C**	**T**	**C**	**C**						
2	-	c	A	c	G	C	-	t	a	t	t	t	G	C	-	-						
3	A	-	-	-	-	-	-	-	-	-	-	-	-	-	t	-						
4	A	-	-	-	-	-	-	-	-	-	-	-	-	-	t	t						
*cmp2*	679	687	688	703	706	707	714	717	720	723	729	731	732	740	761	765	771	774	775	776	912	1107
**1**	**G**	**T**	**G**	**T**	**A**	**A**	**A**	**G**	**C**	**A**	**C**	**C**	**T**	**G**	**A**	**C**	**T**	**T**	**T**	**T**	**C**	**C**
2	-	c	A	c	G	C	t	a	t	t	t	G	C	-	-	-	-	-	-	-	-	-
3	A	-	-	-	-	-	-	-	-	-	-	-	-	-	-	-	-	-	-	-	t	-
4	A	-	-	-	-	-	-	-	-	-	-	-	-	-	G	t	c	c	G	C	-	-
5	A	-	-	-	-	-	-	-	-	-	-	-	-	A	-	-	-	-	-	-	-	t

^a^The corresponding allele numbers were arbitrarily chosen with the reference sequences for *cmp1* and *cmp2* of *C. fetus* subsp. *fetus* strain 82–40 (GenBank acc. No. NC_008599) defined as allele number 1. The hyphen indicates an identical nucelotide as in the reference sequence (allele 1 in bold). Nucleotides in lower case indicate silent mutations

Additionally, the predicted amino acid sequences encoded by *cmp1* and *cmp2* of the reference CFF strain 82–40 were further analyzed. Using the TMB-Hunt program (http://www.bioinformatics.leeds.ac.uk/), we classified both proteins as transmembrane beta barrel proteins using whole sequence amino acid composition [24]. Therefore, the topology of the outer membrane proteins with respect to lipid bilayer was predicted with the PRED-TMBB web server (http://biophysics.biol.uoa.gr/PRED-TMBB/). In the *cmp1* encoded protein, 16 transmembrane domains and 8 external loops were identified, whereas in the *cmp2* encoded protein 20 transmembrane domains and 10 external loops were found. In both, none of the predicted amino substitutions affected the predicted topology of the proteins.

### T4SS and resistance to tetracycline and streptomycin

The T4SS was identified in 11 isolates of cluster A (55 %) and in 1 isolate of cluster B (7 %), in bovine as well as in human samples. There was an expected and complete concordance of the presence of T4SS and resistance to tetracycline and streptomycin, as indicated by higher MIC values. These results suggest that CFF strains with the T4SS all carry a transferable pathogenicity island. Complete genetic analysis and demonstration of the presence of the antibiotic resistance genes *tet(44)* and *ant(6)-Ib* has been shown elsewhere in detail for the bovine clone IMD_523/06 [20].

## Discussion

Human infections with CFF are generally sporadic [6, 25]. Extensive clinical and epidemiological data from our patients infected with CFF show that infection predominantly occurs in the 6^th^ decade and manifests itself mostly as colitis, phlebitis /thrombosis, or infection at sites of foreign bodies such as osteosynthetic or vascular prosthetic material. The patients in this study principally live in the countryside, have regular contacts with farm animals including cattle, horses and pigs, and are consumers of raw meat or raw vegetables. The patients were successfully treated with amoxicillin/clavulanic acid, quinolones, clarithromycin or carbapenems according to susceptibility testing; in the cases of joint prosthesis, only removal of the prosthesis led to cure. No death due to CFF infection occurred in our study.

Our demographic data regarding age at infection (65 % ≥60 years) and source of isolation (blood in 65 %) are in concordance with data from a large survey in Canada reporting age ≥70 years in 53 % and blood as site of infection in 69 % of the cases [13], from France (mean age 69.5 years) [9], or Taiwan (mean age 60.5 years) [11]. It has been suggested that CFF is the most common species causing bloodstream infection among *Campylobacter* species (53 %) [3], and that, as in the present study, patients with CFF infection are more often male [3, 6–12]. Additionally, our data show a larger number of comorbidities and no clear association with liver disease or cirrhosis (only 2 patients with alcohol abuse) [6–12]. Our results confirm the frequent finding of soft tissue infection or thrombosis/thrombophlebitis [6, 9, 10], but, in contrast to one study, colitis was frequent as well [10]. In addition, at variance with previous case reports, we found no infection associated with dialysis procedures [26–28], no infection of the central nervous system [29] or the spine [30–32], no pleural or pulmonary infection [33–35] and no endocarditis [29, 36–39], despite patient 16 carrying an implanted pacemaker (endocarditis was ruled out by repeated transoesophageal echocardiography). Within our study, there were 3 cases of deep muscle abscesses (patients 9 and 15 with psoas abscess, patient 12 with abscess in deep soft tissue of the foot involving the muscles), yet deep muscle abscesses have not been reported previously in the literature. This difference may be due to the low frequency of the infection in the population, the low numbers of patients in the studies, or the physician’s lack of awareness in looking for this infection. A minority of patients in our study were immunosuppressed or immunocompromised, and impairment of the immunological response might favour the occurrence of CFF infection, as does diabetes mellitus and alcohol abuse. Furthermore CFF has a predilection for spreading to sites of implanted medical devices [3, 6, 7, 9, 11]. In contrast to one published report, antibiotic therapy alone failed to eradicate CFF of joint prosthesis infections in our cases. Whether the antibiotic regime applied in the mentioned study (3 weeks of intravenous ceftriaxone followed by a macrolide) was causally responsible for the therapeutic success remains to be established in further cases [40].

Due to the low level of genetic diversity even found in host and geographically unrelated strains, *C. fetus* is considered as a species with a clonal nature [14]. In order to obtain a higher discrimination and to investigate a host preference of CFF isolates, we additionally performed single locus sequence typing (SLST) with the loci of two major outer membrane proteins encoded by *cmp1* and *cmp2*. Low diversity was found, pointing to a conserved basic function of these proteins, or to a low level of adaptive pressure. T4SS genomic islands were found in the groups with the ST-2 and ST-6 sequences only; however, within those groups not in all strains. As there is no correlation between genotypes and the T4SS analysis, T4SS genomic islands seem to spread across strains in a strain-independent manner. When human and bovine isolates were compared, cluster analysis predominantly segregated them into 2 distinct clusters. The level of association between clusters and origin of the isolates was statistically highly significant (*p* < 0.0001), indicating a host preference, with tropism a well known feature of *Campylobacter fetus* subspecies [41]. Within the human samples no clear association was found between the PFGE, MLST or *cmp* types and the clinical findings of the patients.

More striking, however, was the sharing of identical genotypic and phenotypic characteristics within the clusters between strains originating from human and cattle. Despite a host-specific separation in two clusters, the fact that 5 human strains from both clusters were identical to bovine isolates is a strong indication of a possible link between the two hosts. Moreover, the ST-6 found in our study only in human isolates was found in other animal sources as well, mainly cattle [14]. When comparing our data with MLST data of that study based on 140 CFF isolates from diverse geographic backgrounds and host species, we found only a limited number of MLST and *cmp* types, possibly reflecting the circumscribed geographic region in our study. Closer analysis of the mentioned study [14] shows that, in agreement with our findings, there is a larger homology between human and animal strains within the same geographical region than across the globe: for example, the same CFF clone was found in human as well as in bovine samples from Great Britain (C036959 and BT 10/98, respectively). The finding of identical strains, as analyzed by extensive genetic and phenotypic tests, suggests that the same CFF clone can affect both humans and cattle. The fact that only a few strains were found in both human and cattle might indicate animal species not included in this study as sources of infection.

Infection by ingesting raw food has been hypothesized previously [6, 27, 29, 42–45]. In the present study, fifteen out of 17 patients (88 %) had regularly ingested raw food in their diet, with 65 % consuming raw vegetables on a regular basis. Furthermore, 53 % reported a combination of raw food ingestion and the presence of oral ulcers, including wounds due to tooth extraction procedures in patient 1. This latter coincidence has been reported previously [38]. Overall, 82 % reported the presence of skin or mucosal lesions at the time of infection. Whether these observations are pointing to a possible route of infection, as suggested previously [46], remains to be shown.

## Conclusions

Genotypes of CFF showed a certain host association by forming two clusters of mainly human or predominantly bovine isolates. However, the presence of identical strains in humans and cattle may indicate a possible link between strains from these two hosts. Studies with other sources could be addressed in the future.

## Ethics approval and consent to participate

Patient-related clinical information was obtained with written informed consent of patients. The study was approved by the institutional ethics committee at Bern University Hospital and complied with the Declaration of Helsinki.

## Consent for publication

Not applicable.

## Availability of data and materials

The allele sequences of *cmp1* and *cmp2* have been deposited at GenBank under accession numbers KU670818-KU670821 and KU670822-KU670826, respectively. A full matrix including sequences of all strains analyzed in this study can be accessed at http://purl.org/phylo/treebase/phylows/study/TB2:S18853. New alleles and MLST sequence types were deposited in the PubMLST database (http://pubmlst.org/cfetus/).

## Abbreviations

CFF: campylobacter fetus subspecies fetus
CFV: Campylobacter fetus subspecies venerealis
MIC: minimal inhibitory concentration
MLST: multilocus sequence typing
MOMP: major outer membrane protein
PFGE: pulsed-field gel electrophoresis
SLST: single locus sequence typing
subsp.: subspecies
T4SS: type IV secretion system

## References

[1] Skirrow MB (1994). Diseases due to Campylobacter, Helicobacter and related bacteria. J Comp Pathol.

[2] Tremblay C, Gaudreau C (1998). Antimicrobial susceptibility testing of 59 strains of Campylobacter fetus subsp. fetus. Antimicrob Agents Chemother.

[3] Gazaigne L, Legrand P, Renaud B, Bourra B, Taillandier E, Brun-Buisson C, Lesprit P (2008). Campylobacter fetus bloodstream infection: risk factors and clinical features. Eur J Clin Microbiol Infect Dis.

[4] Hagiya H, Matsumoto M, Furukawa H, Murase T, Otsuka F (1933). Mycotic Abdominal Aortic Aneurysm Caused by Campylobacter fetus: A Case Report and Literature Review. Ann Vasc Surg.

[5] Morrison VA, Lloyd BK, Chia JK, Tuazon CU (1990). Cardiovascular and bacteremic manifestations of Campylobacter fetus infection: case report and review. Rev Infect Dis.

[6] Wagenaar JA, van Bergen MA, Blaser MJ, Tauxe RV, Newell DG, van Putten JP (2014). Campylobacter fetus infections in humans: exposure and disease. Clin Infect Dis.

[7] Cypierre A, Denes E, Barraud O, Jamilloux Y, Jacques J, Durox H, Pinet P, Weinbreck P (2014). Campylobacter fetus infections. Med Mal Infect.

[8] Bessède E, Lehours P, Labadi L, Bakiri S, Mégraud F (2014). Comparison of characteristics of patients infected by Campylobacter jejuni, Campylobacter coli, and Campylobacter fetus. J Clin Microbiol.

[9] Pacanowski J, Lalande V, Lacombe K, Boudraa C, Lesprit P, Legrand P, Trystram D, Kassis N, Arlet G, Mainardi JL, Doucet-Populaire F, Girard PM, Meynard JL, CAMPYL Study Group (2008). Campylobacter bacteremia: clinical features and factors associated with fatal outcome. Clin Infect Dis.

[10] Pigrau C, Bartolome R, Almirante B, Planes AM, Gavalda J, Pahissa A (1997). Bacteremia due to Campylobacter species: clinical findings and antimicrobial susceptibility patterns. Clin Infect Dis.

[11] Liao CH, Chuang CY, Huang YT, Lee PI, Hsueh PR (2012). Bacteremia caused by antimicrobial resistant Campylobacter species at a medical center in Taiwan, 1998–2008. J Infect.

[12] Saito S, Naito T, Kukino J, Okumura T, Sekiya S, Isonuma H, Watanabe K, Dambara T, Hayashida Y (2004). Campylobacter fetus subsp. fetus sepsis: a case report and review of the literatures in Japan. Kansenshogaku Zasshi.

[13] Tremblay C, Gaudreau C, Lorange M (2003). Epidemiology and antimicrobial susceptibilities of 111 Campylobacter fetus subsp. fetus strains isolated in Québec, Canada, from 1983 to 2000. J Clin Microbiol.

[14] van Bergen MA, Dingle KE, Maiden MC, Newell DG, van der Graaf-Van Bloois L, van Putten JP, Wagenaar JA (2005). Clonal nature of Campylobacter fetus as defined by multilocus sequence typing. J Clin Microbiol.

[15] Hurcombe SD, Fox JG, Kohn CW (2009). Isolation of Campylobacter fetus subspecies fetus in a two-year-old quarterhorse with chronic diarrhea of an undetermined etiology. J Vet Diagn Invest.

[16] Sprenger H, Zechner EL, Gorkiewicz G (2012). So close and yet so far – Molecular Microbiology of Campylobacter fetus subspecies. Eur J Microbiol Immunol.

[17] Cody AJ, Maiden MJ, Dingle KE (2009). Genetic diversity and stability of the porA allele as a genetic marker in human Campylobacter infection. Microbiology.

[18] Cascales E, Christie PJ (2003). The versatile bacterial type IV secretion systems. Nat Rev Microbiol.

[19] Juhas M (2015). Horizontal gene transfer in human pathogens. Crit Rev Microbiol.

[20] Abril C, Brodard I, Perreten V (2010). Two novel antibiotic resistance genes, tet(44) and ant(6)-Ib, are located within a transferable pathogenicity island in Campylobacter fetus subsp. fetus. Antimicrob Agents Chemother.

[21] Abril C, Vilei EM, Brodard I, Burnens A, Frey J, Miserez R (2007). Discovery of insertion element ISCfe1: a new tool for Campylobacter fetus subspecies differentiation. Clin Microbiol Infect.

[22] Fujita M, Fujimoto S, Morooka T, Amako K (1995). Analysis of strains of Campylobacter fetus by pulsed-field gel electrophoresis. J Clin Microbiol.

[23] Dworkin J, Tummuru MK, Blaser MJ (1995). Segmental conservation of sapA sequences in type B Campylobacter fetus cells. J Biol Chem.

[24] Garrow AG, Agnew A, Westhead DR (2005). TMB-Hunt: a web server to screen sequence sets for transmembrane beta-barrel proteins. Nucleic Acids Res.

[25] Harvey S, Greenwood JR (1985). Isolation of *Campylobacter fetus* from a pet turtle. J Clin Microbiol.

[26] Romero Gómez MP, García-Perea A, Ruiz Carrascoso G, Bajo MA, Mingorance J (2010). Campylobacter fetus peritonitis and bacteremia in a patient undergoing continuous ambulatory peritoneal dialysis. J Clin Microbiol.

[27] Shimizu Y, Ishii A, Takahata A, Kajiyama T, Yamahatsu A, Io H, Kurusu A, Hamada C, Horikoshi S, Tomino Y (2012). Campylobacter bacteremia in hemodialysis patients by eating raw meat - the importance of sanitary education. Case Rep Nephrol Urol.

[28] Lee YC, Huang YT, Sheng WH, Hsueh PR (2011). Simultaneous peritoneal dialysis-associated peritonitis and bacteremia due to ceftriaxone-resistant Campylobacter fetus. Perit Dial Int.

[29] Sui F, Le Dû D, Roux AL, Hanachi M, Dinh A, Crémieux AC (2013). Meningitis and endocarditis caused by Campylobacter fetus after raw-liver ingestion. J Clin Microbiol.

[30] Tanaka A, Takahashi J, Hirabayashi H, Ogihara N, Mukaiyama K, Shimizu M, Hashidate H, Kato H (2012). A Case of Pyogenic Spondylodiscitis Caused by Campylobacter fetus for Which Early Diagnosis by Magnetic Resonance Imaging Was Difficult. Asian Spine J.

[31] Chaillon A, Baty G, Lauvin MA, Besnier JM, Goudeau A, Lanotte P (2010). Campylobacter fetus subspecies fetus spondylodiscitis. J Med Microbiol.

[32] Wong JSJ, Anderson TP, Chambers ST, On SLW, Murdoch DR (2009). Campylobacter fetus-associated epidural abscess and bacteremia. J Clin Microbiol.

[33] Alnimr AM (2014). A case of bacteremia caused by Campylobacter fetus: an unusual presentation in an infant. Infect Drug Resist.

[34] Yamagami K, Miyashita T, Nakamura T, Shirano M, Nakamura T, Kameda K, Nishijima M, Imanishi M, Yang X, Kanegane H (2014). Campylobacter fetus bacteremia with purulent pleurisy in a young adult with primary hypogammaglobulinemia. Intern Med.

[35] Decousser JW, Prouzet-Mauléon V, Bartizel C, Gin T, Colin JP, Fadel N, Holler C, Pollet J, Megraud F (2007). Fatal relapse of a purulent pleurisy caused by Campylobacter fetus subsp. fetus. J Clin Microbiol.

[36] Désidéri-Vaillant C, Guichon JM, Noyer V, Nedelec Y, Galinat H, Sapin-Lory J, Di Costanzo L, Le Guen P, Nicolas X (2013). [Campylobacter fetus endocarditis: a case report]. Ann Biol Clin.

[37] Haruyama A, Toyoda S, Kikuchi M, Arikawa T, Inami S, Otani N, Amano H, Matsuda R, Inoue T (2011). Campylobacter fetus as cause of prosthetic valve endocarditis. Tex Heart Inst J.

[38] Miki K, Maekura R, Hiraga T, Hirotani A, Hashimoto H, Kitada S, Miki M, Yoshimura K, Naka N, Motone M, Fujikawa T, Takashima S, Kitazume R, Kanzaki H, Nakatani S, Watanuki H, Tagusari O, Kobayashi J, Ito M (2005). Infective tricuspid valve endocarditis with pulmonary emboli caused by Campylobacter fetus after tooth extraction. Intern Med.

[39] Ledina D, Ivic I, Karanovic J, Karanovic N, Kuzmicic N, Ledina D, Puljiz Z (2012). Campylobacter fetus infection presenting with bacteremia and cellulitis in a 72-year-old man with an implanted pacemaker: a case report. J Med Case Rep.

[40] Chambers ST, Morpeth SC, Laird HM (2005). Campylobacter fetus prosthetic hip joint infection: successful management with device retention and review. J Infection.

[41] Kienesberger S, Sprenger H, Wolfgruber S, Halwachs B, Thallinger GG, Perez-Perez GI, Blaser MJ, Zechner EL, Gorkiewicz G (2014). Comparative genome analysis of Campylobacter fetus subspecies revealed horizontally acquired genetic elements important for virulence and niche specificity. PLoS One.

[42] Inoue K, Kitamura H, Nagasawa Y, Kawada N, Isaka Y, Rakugi H (2010). Campylobacter fetus peritonitis in a patient with an unused embedded subcutaneous peritoneal catheter. Perit Dial Int.

[43] Serraino A, Florio D, Giacometti F, Piva S, Mion D, Zanoni RG (2013). Presence of Campylobacter and Arcobacter species in in-line milk filters of farms authorized to produce and sell raw milk and of a water buffalo dairy farm in Italy. J Dairy Sci.

[44] Kramer JM, Frost JA, Bolton FJ, Wareing DR (2000). Campylobacter contamination of raw meat and poultry at retail sale: identification of multiple types and comparison with isolates from human infection. J Food Prot.

[45] Ichiyama S, Hirai S, Minami T, Nishiyama Y, Shimizu S, Shimokata K, Ohta M (1998). Campylobacter fetus subspecies fetus cellulitis associated with bacteremia in debilitated hosts. Clin Infect Dis.

[46] Martinez-Balzano C, Kohlitz PJ, Chaudhary P, Hegazy H (2013). Campylobacter fetus bacteremia in a young healthy adult transmitted by *khat* chewing. J Infection.

